# Potential Harms of Ocular and Periorbital Topical Vitamin A Application: A Systematic Review and Meta-Analysis

**DOI:** 10.1167/iovs.67.11.11

**Published:** 2026-09-08

**Authors:** Kaleb S. Abbott, Zanna Kruoch, Andrew Pucker, Anna A. Tichenor, Matt Orgill, Brooke Anderson, Erica Olsen, Joshua Dusek, Daniel Shaughnessy, Kevin Naaman, Riaz Qureshi

**Affiliations:** 1 Department of Ophthalmology, University of Colorado Denver Anschutz Medical Campus, Aurora, Colorado, United States; 2 College of Optometric Medicine, Rocky Mountain University of Health Professions, Provo, Utah, United States; 3 Eminent Ophthalmic Services, LLC, Milledgeville, Georgia, United States; 4 School of Optometry, Indiana University, Bloomington, Indiana, United States; 5 Department of Epidemiology, Colorado School of Public Health, Aurora, Colorado, United States

**Keywords:** vitamin A, ocular surface, adverse events, topical, systematic review, meta-analysis

## Abstract

**Purpose:**

Topical vitamin A derivatives (retinoids) are widely used for dermatologic and cosmetic indications and are often applied to the face and periorbital region. Although vitamin A is essential for epithelial integrity and tear film homeostasis, concerns exist regarding potential adverse effects on the ocular surface and meibomian glands. This systematic review and meta-analysis evaluated ophthalmic and periorbital harms associated with topical vitamin A products.

**Methods:**

PubMed, Web of Science, Scopus, and the Cochrane Library were searched from inception to August 25, 2025, following PRISMA guidelines (PROSPERO CRD42024523189). Randomized and nonrandomized human studies evaluating topical vitamin A products applied to the ocular surface or periorbital region were included. Studies were independently screened, data were extracted, and risk of bias was assessed by two reviewers. Dichotomous harms were pooled using Peto fixed-effects odds ratios (ORs) with 95% confidence intervals (CIs).

**Results:**

Eighty-nine studies (10,794 participants) met inclusion criteria; 55 were included in meta-analysis. Compared with controls, topical vitamin A derivatives increased odds of pain (OR = 6.37), dryness (OR = 2.69), burning (OR = 2.44), and any harm (OR = 1.71). Serious adverse events were uncommon and not significantly increased (OR = 1.21). Prescription-strength products showed higher odds of harm, and non-prescription formulations were not significantly associated with increased harms. Evidence certainty ranged from very low to low.

**Conclusions:**

Topical vitamin A products applied to the ocular or periorbital region are associated with increased ocular discomfort and surface-related harms, particularly with prescription-strength formulations, with important implications for dry eye and meibomian gland dysfunction management.

Vitamin A (vit A) is an essential fat-soluble micronutrient used in topical and oral forms to support skin health, vision, immune function, and cellular growth.^1^ Topical vit A derivatives (retinoids, such as retinyl palmitate, retinoic acid, and tretinoin) are widely used in dermatology to treat acne, reduce age-related skin changes, and improve skin texture by stimulating collagen production.^2^ These agents are available in over-the-counter (OTC) and prescription formulations, with potency primarily determined by conversion to retinoic acid.^1^^,^^3^^,^^4^ Retinyl esters are sequentially converted to retinol, retinaldehyde, and ultimately retinoic acid.^5^ Retinoic acid mediates therapeutic effects by binding retinoic acid receptors on epithelial cells, reducing sebaceous gland activity, modulating inflammatory pathways, and normalizing keratinocyte proliferation and differentiation.^2^^,^^6^^–^^8^

**Table:** 

Retinoid	Form	Strength	Availability
Synthetic retinoids (e.g., isotretinoin, adapalene, tazarotene)	Analogs with receptor selectivity and enhanced potency	Highest	Prescription
Retinoic acid (tretinoin)	Active form	High	Prescription
Retinaldehyde (retinal)	One-step conversion to retinoic acid	Medium	Over the counter
Retinol	Two-step conversion to retinoic acid	Low	Over the counter
Retinyl esters (e.g., retinyl palmitate, retinyl acetate, retinyl linoleate)	Three-step conversion to retinoic acid	Weak	Over the counter
β-Carotene (PROVIT-A)	Converted in the body to retinal	Weakest	Dietary source or supplement

Within the lacrimal glands, vit A is stored as retinyl esters and secreted into the tear film primarily as retinol, which is then metabolized into retinoic acid by ocular surface epithelial cells.^9^^,^^10^ On the ocular surface, vit A supports epithelial differentiation, promotes corneal wound healing, maintains balanced keratinization, and contributes to mucin production (e.g., MUC5AC), all of which are critical for tear film stability.^11^^–^^17^ Conversely, abnormal endogenous or exogenous vit A levels may also worsen dry-eye disease (DED).^12^^,^^18^^–^^20^

The meibomian glands (MGs) are specialized sebaceous glands that secrete meibum, a lipid-rich substance essential for tear film stability.^21^ Because vit A reduces sebum production, periorbital application—particularly of 13-*cis* retinoic acid—may adversely affect MG structure and function.^2^^,^^4^^,^^22^^,^^23^ Oral isotretinoin induces MG atrophy and reduces meibum secretion, and in vivo studies have shown that 13-*cis* retinoic acid upregulates keratinization-related genes in MGs, inhibiting epithelial proliferation and promoting gland atrophy.^22^^–^^25^ These changes may contribute to meibomian gland dysfunction (MGD) and exacerbate DED.^25^

## Rationale for This Review

Since 1955, more than 2000 vit A derivatives have been synthesized for dermatological use.^26^ Meanwhile, DED prevalence continues to rise.^27^ Although less-potent ophthalmic vit A formulations have been reported to benefit ocular surface disease,^11^ there is no consensus regarding ocular effects, dosing, or dose–response relationships of oral or topical vit A products. In particular, the threshold at which vit A derivatives may induce MGD or cause eye irritation, potentially offsetting dermatologic therapeutic benefit, remains unclear. To date, no systematic review has comprehensively evaluated the adverse effects of ophthalmic or periorbital vit A formulations on the ocular surface or MGs, making clarification of their potential harms relevant for dermatologists who prescribe or recommend these products, as well as eye-care providers managing MGD and DED.

This systematic review evaluates the potential ophthalmic and periorbital harms of topical vit A–containing products, with an emphasis on MG viability, function, and DED signs and symptoms. Secondarily, it assesses additional adverse effects associated with their use. In this review, the term “vit A derivatives” refers to active retinoid compounds (e.g., retinol, retinaldehyde, retinoic acid, retinyl esters), whereas the term “vit A products” refers to topical formulations containing one or more of these derivatives.

## Methods

### Search Strategy

This review was registered on PROSPERO (CRD42024523189), and the protocol was posted on the Open Science Framework (https://osf.io/saq7n/).^28^ The protocol also proposed a methodological study within a review to assess the accuracy of at least one large language model for screening and data extraction tasks. That sub-study will be presented elsewhere; this review focuses on the potential harms of topical vit A. Supplemental Appendix A in the online supplementary material provides a complete description of the methods; an overview is provided below. The MOOSE checklist is also provided in the online Supplementary Material (Supplementary Material: ISSM_MOOSE_Checklist_VitaminAHarms).

Four databases were searched, supplemented with snowball searching of included studies, from inception to August 25, 2025, with no restrictions on language. The search strategies are provided in Supplementary Appendix A. Records were imported into PICO Portal for deduplication and screening by two reviewers. Title and abstract screening was stopped when PICO Portal artificial intelligence (AI) algorithms reached an acceptable threshold of accuracy for ruling out remaining records (i.e., 98% negative predictive value).

### Inclusion and Exclusion Criteria

Studies of any design that assessed the application of vit A products topically to the eye (e.g., through eye drops or ointment), eyelids, or the periorbital area for any indication, in any population, and with any comparator were included. Eligibility was not restricted based on systemic or dermatologic comorbidities. Studies involving participants with concurrent use of eye drops known to induce DED signs or symptoms (e.g., glaucoma drops), studies with confounding interventions or confounding factors (e.g., use of dry eye treatments), and animal or preclinical studies were excluded.

### Outcome Measures and Evaluation Criteria

The primary outcome was the occurrence of ophthalmic or periorbital harms associated with topical vit A application. Outcome data extracted included specific harms (pain, dryness, burning, redness, stinging, itching, edema, scaling, skin damage, serious harms, and any reported harm), as well as reported ocular surface outcomes, including dry eye symptoms, corneal and conjunctival staining, and MG parameters. The critical outcome was the overall prevalence of specific harms to the eye or face over the course of follow-up.

### Study Selection and Data Extraction

Data were extracted by one reviewer and independently by a second reviewer using a structured form within PICO Portal. Study characteristics, participant characteristics, and outcome data were extracted. For multi-arm studies, data relevant to the intervention and comparator groups were extracted. For studies that reported results at multiple time points, data were extracted for the latest time point.

### Risk of Bias Assessment

Two review authors independently assessed the risk of bias (RoB) using one of two tools, as relevant to the study type: Cochrane's RoB 2.0 for randomized controlled trials (RCTs)^29^ and a modified Newcastle Ottawa Scale for all other study types.^30^

### Statistical Analysis

Analyses were conducted in accordance with Chapter 10 of the *Cochrane Handbook*.^31^ For dichotomous harms outcomes, Peto one-step fixed-effects odds ratio (OR) meta-analyses were conducted. For prevalence harms outcomes, random-intercept logistic regression models with 95% CIs, 95% prediction intervals (PIs), and the maximum-likelihood estimator for τ² were used.^32^^,^^33^ A subgroup analysis was performed using the Peto OR of any harm and a binary indicator for vit A derivative strength (i.e., high-strength prescription vs. low or moderate-strength non-prescription).

### Certainty of Evidence

A summary of findings table was prepared to assess the certainty of evidence following the guidelines of Chapter 14 of the *Cochrane Handbook*.^34^ Specifically, two review authors independently applied the Grading of Recommendations for Assessment, Development, and Evaluation (GRADE) framework to rate overall certainty as “high,” “moderate,” “low,” or “very low.”^34^ Certainty of the body of evidence was downgraded if any of the following were substantially present in the contributing studies to an outcome: (1) risk of bias, (2) heterogeneity/inconsistency, (3) imprecision, (4) indirectness, and (5) publication bias. Very large effects were also considered as a possible reason for upgrading the certainty of evidence, as some of the evidence came from non-randomized studies.^34^

## Results

**Figure 1.**
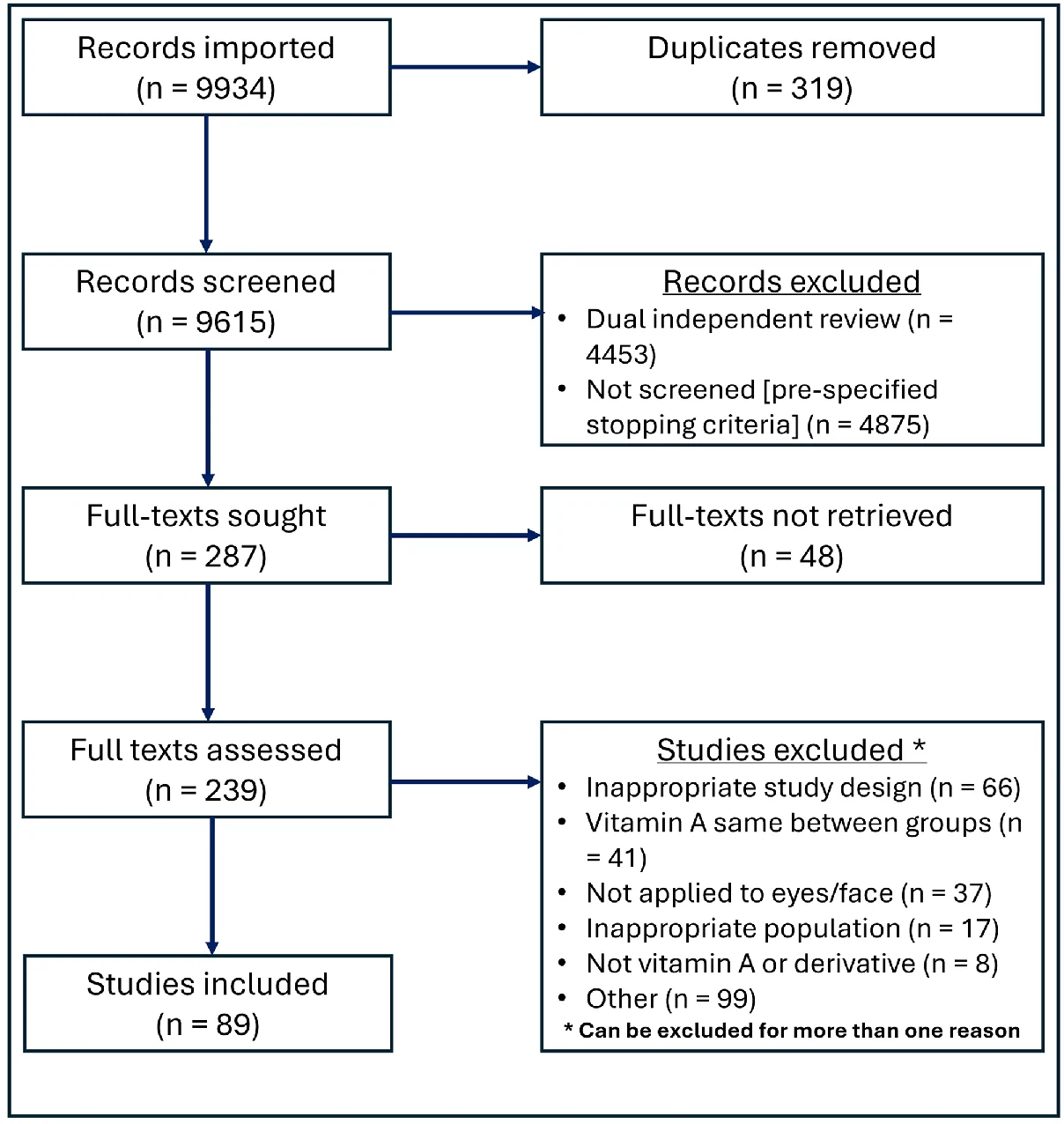
Study flow diagram. PRISMA, Preferred Reporting Items for Systematic Reviews and Meta-Analyses.

The search yielded 9649 unique titles and abstracts. Given the data on predictions provided in PICO Portal, title-abstract screening continued until at least 98% of predicted includes had been captured, with at least 98% negative predictive value (NPV), thereby indicating a high likelihood that the remaining records were accurately predicted as excluded. A total of 4745 titles and abstracts were screened before screening was ceased when PICO Portal predicted that over 99% of eligible records had been included. PICO Portal predictions (based on 4741 records) included 92 true positives, 1130 false positives, 0 false negatives, and 3519 true negatives, giving a NPV of 100%. After screening 292 full-text reports, a total of 89 studies were included (Fig. 1).

### Characteristics of Included Studies

#### Types of Studies

Eighty-nine studies were included, of which 11 (12%) were identified as only registry listings with no results available and 18 (20%) were abstracts with no accompanying full-text publication. Randomized controlled trials were the predominant study design (*n* *=* 67, 75%), with fewer single-arm before–after studies (*n* *=* 13, 15%) and cohort studies (*n* *=* 9, 10%). The United States contributed the most participants (*n* *=* 21, 24%), followed by China (*n* *=* 12, 13%). Nineteen studies (21%) did not report study location. A high percentage of studies were funded by industry (*n* *=* 43, 48%), and 31 trials (35%) did not report their source of funding. Most trials were conducted in specialized hospital and clinic settings (*n* *=* 45, 51%), and most studies were of short duration, with 60 (67%) lasting less than 3 months and 26 (29%) lasting 3 to 12 months; only one study extended beyond 1 year. Table 1 presents a summary of the characteristics of included studies, and specific details by study can be found in Supplementary Table S1.

**Table 1.** Characteristics of Included Studies

	Distribution *n* (%)
Study Characteristics (*n* = 89)	
Study design	
RCT	67 (75)
Single arm–before/after	13 (15)
Cohort	9 (10)
Country	
United States	21 (24)
China	12 (13)
India	6 (7)
France	5 (6)
South Korea	5 (6)
United Kingdom	4 (4)
Iran	4 (4)
Canada	3 (3)
Japan	3 (3)
Indonesia	3 (3)
Germany	2 (2)
Australia	2 (2)
Other (Austria, Brazil, Lebanon, New Zealand, Norway, Pakistan, Romania, Spain, Sri Lanka, Sweden, Turkey)	12 (13)
Not reported	19 (21)
Setting	
Specialized hospital/clinics	45 (51)
Primary care hospital/general clinic	4 (4)
Not reported	40 (45)
Sources of funding	
Industry	43 (48)
Government	5 (6)
Institution/organization	5 (6)
Explicitly “no funding”	5 (6)
Foundation/charity	2 (2)
Not reported	31 (35)
Duration of trial	
Immediate (<1 mo)	12 (13)
1 to <3 mo	48 (54)
3 to <12 mo	26 (29)
>12 mo	1 (1)
Reported ocular surface outcomes	
No	72 (81)
Yes	17 (19)
Corneal staining	14 (16)
Dry eye symptoms	13 (15)
Conjunctival staining	3 (3)
Meibomian glands	2 (2)
Schirmer's	1 (1)
Statements on harms	
Specifies which harms occurred	52 (58)
Explicit statement that no harms occurred, specify which harms did not occur	3 (3)
Explicit statement that no harms occurred, does not specify which harms did not occur	8 (9)
Study does not report any harms data^*^	26 (29)
Harms data useable in meta-analyses	
Yes	56 (63)
No^*^	33 (37)
Location of harms	
Skin	19 (21)
Ocular	15 (17)
Ocular and skin	6 (7)
Not reported/not specified^*^	49 (55)
Arm/Intervention Characteristics (*n =* 181)	
Vitamin A derivative	
Tretinoin/retin-A/retinoic acid	34 (19)
Retinol	23 (13)
Retinyl palmitate/propionate/acetate	13 (7)
Retinaldehyde (retinal)	5 (3)
Tazarotene	5 (3)
Adapalene	2 (1)
Isotretinoin	1 (1)
Other (e.g., non-specific vit A)	20 (11)
Non–vit A control (e.g., placebo)	78 (43)
Site of application	
Face (periorbital not specified)	104 (57)
Face (periorbital specified)	20 (11)
Eye or eyelid	53 (30)
Not reported	4 (2)
Type of product	
Cream/moisturizer	73 (40)
Eye drop	29 (16)
Ointment	15 (8)
Ophthalmic gel	9 (5)
Facial gel	13 (7)
Other (e.g., emulsion, serum)	36 (20)
Not reported	5 (3)

* Includes registries that are ongoing and do not have results

#### Types of Participants

A total of 10,794 participants were enrolled in the included studies. Across the 45 studies that reported ages of participants, the ages ranged from 18 to 70 years, with a nearly even split across studies that assessed younger than 40 years (*n* *=* 20) and older than 40 years (*n* *=* 25). Most studies predominantly enrolled females, with 40 enrolling larger proportions of women (i.e., more than two thirds) than men, 12 enrolling approximately equal proportions of women and men, and only three enrolling larger proportions of men than women. Most studies enrolled adults undergoing treatment for dermatologic conditions such as acne, photoaging, or cosmetic concerns, and a minority (*n* *=* 17, 19%) were evaluated for ocular surface diseases.

#### Types of Interventions

There were 181 treatment and control arms with widely varying types of vit A derivatives. Tretinoin or retinoic acid was the most investigated (*n* *=* 34, 19%), followed by retinol (*n* *=* 23, 13%) and retinyl esters (e.g., palmitate, propionate, or acetate) (*n* *=* 13, 7%). In 20 studies (11%), the vit A product was mixed or unspecified. Nearly half of all study arms included a non–vit A comparator (*n* *=* 78, 43%), most often a vehicle placebo. Other non–vit A controls included no treatment, oils (e.g., soy, peanut), other active ointments and gels (e.g., lubratex, cyclosporine A, tobramycin eye drops, sodium hyaluronate), or completely different approaches to treatment such as microneedling or moisture chambers (Supplementary Table S1).

The site and method of application also varied. Application to the face without further specification was the most common (*n* *=* 104, 57%), with an additional 20 arms (11%) specifically including the periorbital region and 53 (30%) specifying direct ocular administration. Creams or moisturizers were the most common type of product (*n* *=* 73, 40%), followed by eye drops (*n* *=* 29, 16%), ointments (*n* *=* 15, 8%), and facial (*n* *=* 13, 7%) or ophthalmic (*n* *=* 9, 5%) gels.

#### Types of Outcomes

Most studies (*n* = 72, 81%) did not report ocular surface outcomes. The most reported ocular surface outcomes were corneal staining (*n* = 14, 16%) and DED symptoms (*n* = 13, 15%), with less frequent assessment of conjunctival staining (*n* = 3, 3%) and MG parameters (*n* = 2, 2%), such as meibum secretion scores and meibography.

### RoB Assessment

**Figure 2.**
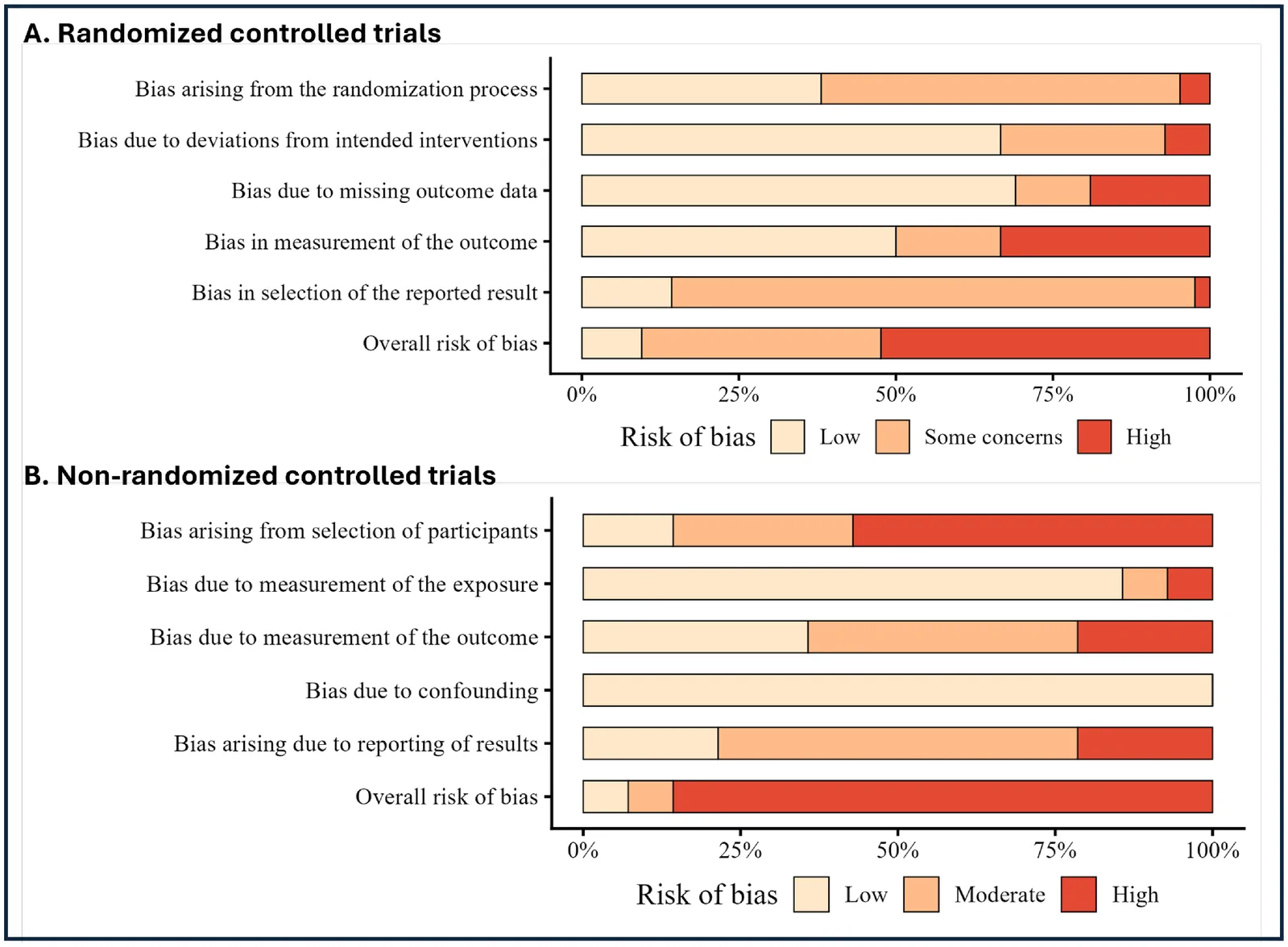
(**A**) RoB for 42 randomized controlled trials assessed using the Cochrane RoB 2.0 tool. (**B**) RoB for 14 non-randomized studies assessed using a modified Newcastle–Ottawa Scale.

Of the 89 studies, RoB was not assessed for 33 due to limited data (i.e., no full texts) or lack of any data for harms. Summaries of RoB judgments for the 42 RCTs and 14 non-RCTs are reported in Supplementary Table S2 and Figures 2A and 2B. For the 42 RCTs, overall RoB assessments showed that four (10%) were judged to be at low RoB, 16 (38%) had some concerns, and 22 (52%) had high RoB. Common sources of bias across RCTs were inadequate reporting of how the randomization sequence was generated, poor measurement of the outcome (i.e., harms), and lack of detail (or absence of) protocols and registrations.

For the 14 non-RCTs, overall RoB assessments showed that one (7%) was judged to be at low RoB, one (7%) had moderate RoB, and 12 (86%) had high RoB. Most of the non-RCTs had RoB arising from selection of participants, measurement of the outcome, and reporting of results. A majority of the non-RCTs had low RoB due to measurement of the exposure being clearly described. Additionally, given that the studies merely reported frequencies of harms and did not conduct an inferential analysis for harms, all studies were judged to be at low risk of bias due to confounding for analysis.

### Synthesis of Results

#### Harms Reporting

The reporting of harms varied substantially across studies. Of the 89 included studies, 63 (71%) reported some form of statement on harms. Among these, 52 (83%) specified which harms occurred, three (5%) explicitly stated that no harms occurred and listed which events were absent, and eight (13%) stated only that no harms occurred without further specification. Usable data for meta-analysis were provided by 55 (87%) studies. The remaining 26 studies (29%) did not report harms data, although these included the registries for which no data were available.

#### Specific Harms

Forty-nine studies (55%) did not differentiate whether events were ocular or dermatologic. Among the 49 studies that reported harms without clear differentiation between ocular and dermatologic outcomes, 19 (39%) reported only skin-related harms, 15 (31%) reported ocular harms, and six (12%) reported both ocular and skin harms. Most harms were described as mild to moderate, transient, and self-limiting. Some reports described persistent ocular surface discomfort, eyelid inflammation, or light sensitivity, particularly in studies examining periorbital applications. Very few studies assessed MG function, limiting the ability to determine the effect of topical vit A on MGD.

Table 2 presents the results of all meta-analyses for harms; Supplementary Figures S1 to S24 present the forest plots for all Peto OR meta-analyses and random-intercept logistic regression meta-analyses. Across all vit A interventions, the ocular or periorbital harms with the largest ORs were pain, dryness, and burning. Pooled estimates demonstrated significantly increased odds of these events compared with non–vit A controls: generalized pain OR = 6.37 (95% confidence interval [CI], 4.47–9.08; *I*^2^ = 45%; 10 studies, 2914 participants; very low certainty evidence); dryness OR = 2.69 (95% CI, 2.11–3.42; *I*^2^ = 74%; 12 studies, 3281 participants; low certainty evidence); and burning OR = 2.44 (95% CI, 1.68–3.56; *I*^2^ = 0%; eight studies, 1600 participants; low certainty evidence). Pooled analyses showed elevated odds of general occurrence of any harm (OR = 1.71; 95% CI, 1.45–2.02; *I*^2^ = 77%; 15 studies, 3579 participants; very low certainty evidence). Serious harms were rare and had low odds of an association with vit A derivatives compared with controls (OR = 1.21; 95% CI, 0.53–2.76; *I*^2^ = 0%; six studies, 3099 participants; very low certainty evidence). Table 3 presents a summary of findings and GRADE assessments for key outcomes.

**Table 2.** Meta-Analyses of Harms

	Peto Odds Ratios			Random Intercept Logistic Regression		
Adverse Event	Studies (Participants), *n*	OR	95% CI	Studies (Participants), *n*	*P*	(95% CI) [95% PI]
Pain	10 (2914)	6.37	(4.47–9.08)	11 (1653)	0.25	(0.07–0.61) [<0.01–0.99]
Dryness	12 (3281)	2.69	(2.11–3.42)	15 (1892)	0.14	(0.06–0.27) [0.01–0.84]
Burning	8 (1600)	2.44	(1.68–3.56)	12 (1321)	0.05	(0.02–0.12) [<0.01–0.69]
Scaling	5 (1318)	1.98	(1.45–2.71)	6 (829)	0.15	(0.08–0.25) [0.03–0.46]
Itchiness	10 (1585)	1.78	(1.26–2.51)	12 (979)	0.13	(0.08–0.20) [0.02–0.47]
Stinging	3 (982)	1.78	(0.96–3.32)	6 (712)	0.03	(0.01–0.09) [<0.01–0.24]
Redness	13 (3329)	1.29	(1.02–1.64)	19 (1907)	0.06	(0.02–0.16) [<0.01–0.83]
Skin damage	6 (1094)	1.52	(1.13–2.03)	7 (694)	0.12	(0.02–0.46) [<0.01–0.98]
Edema	3 (168)	1.21	(0.51–2.85)	6 (122)	0.04	(<0.01–0.32) [<0.01–0.96]
Serious AEs	6 (3099)	1.21	(0.53–2.76)	13 (2261)	<0.01	(<0.01–0.01) [<0.01–0.02]
Other harms^*^	17 (2007)	1.58	(1.09–2.30)	26 (2866)	0.06	(0.03–0.11) [<0.01–0.64]
Any harm	15 (3579)	1.71	(1.45–2.02)	38 (3021)	0.03	(0.01–0.12) [<0.01–0.99]

* Other harms include withdrawal due to harm, foreign body sensation, eye irritation, conjunctivitis, corneal ulcer, tightness, prickling, drug allergy, systemic side effects, and combinations of specific harms.

**Table 3.** GRADE Summary of Findings

Outcome	Estimate (95% CI)	Participants (Studies), *n*	Certainty of the Evidence	Conclusions
Relative odds of any skin and/or ocular harms by the latest time pointRisk (proportion) of any skin and/or ocular harms by the latest time point	OR = 1.71 (1.45–2.02)Proportion = 0.03 (0.01–0.12)	3579 (15 RCTs and non-RCTs)3021 (38 RCTs and non-RCTs)	⊕⊖⊖⊖ Very low^*^^,^^†^^,^^‡^⊕⊖⊖⊖Very low^*^^,^^†^^,^^§^	Vitamin A derivatives may increase the odds of any skin and/or ocular harms, but the evidence is very uncertain
Relative odds of serious adverse events by the latest time pointRisk (proportion) of any serious adverse events by the latest time point	OR = 1.21 (0.53–2.76)Proportion = 0.00 (0.00–0.01)	3099 (6 RCTs)2261 (13 RCTs)	⊕⊖⊖⊖Very low^||^^,^^¶^^,^^#^⊕⊕⊖⊖Low^||^^,^^#^	Vitamin A derivatives may have little or no effect on the odds of serious adverse events
Relative odds of pain by the latest time point	OR = 6.37 (4.47–9.08)	2914 (10 RCTs and non-RCTs)	⊕⊖⊖⊖Very low^*^^,^^†^^,^^‡^	Vitamin A derivatives may increase the odds of pain, but the evidence is very uncertain
Relative odds of dryness by the latest time point	OR = 2.69 (2.11–3.42)	3281 (12 RCTs)	⊕⊕⊖⊖Low^†^^,^^‡^^,^^||^	Vitamin A derivatives may result in a large increase in the odds of dryness
Relative odds of burning by the latest time point	OR = 2.44 (1.68–3.56)	1600 (8 RCTs)	⊕⊕⊖⊖Low^†^^,^^‡^^,^^||^	Vitamin A derivatives may result in a large increase in the odds of burning

The population included people with any characteristics who applied topical vit A derivatives in any form to the face, periorbital area, or eyes directly. The setting included specialized clinics under controlled circumstances, primarily participant blinded, industry-funded, RCTs. Interventions included any vit A derivative product (prescription and non-prescription), such as creams, ointments, or drops. Comparators included non–vit A controls, primarily placebo or vehicle controls.

* Downgraded two levels for RoB: mostly high RoB studies.

† Downgraded two levels for publication bias: history of vit A derivative development and use, ease-of-access (non-prescription), and commonly compounded and integrated into other products. Known concerns included inconsistency and incompleteness of mild/general harms collection and reporting.

‡ Upgraded one level for large effect: statistical and consistent evidence of a large increase in the odds of harm.

§ Downgraded one level for inconsistency. Most studies showed very low overall risk (18/35 had 0%, 4/35 had <10%), but 12/35 had risks between 20% and 100%.

|| Downgraded one level for RoB: mix of low RoB, some concerns, and high RoB studies.

¶ Downgraded one level for imprecision: 95% CIs ranged from half the odds to twice the odds.

# Downgraded one level for publication bias: similar problems with publication history of vit A derivatives as above, but serious adverse events were less likely to be not reported in existing publications.

**Figure 3.**
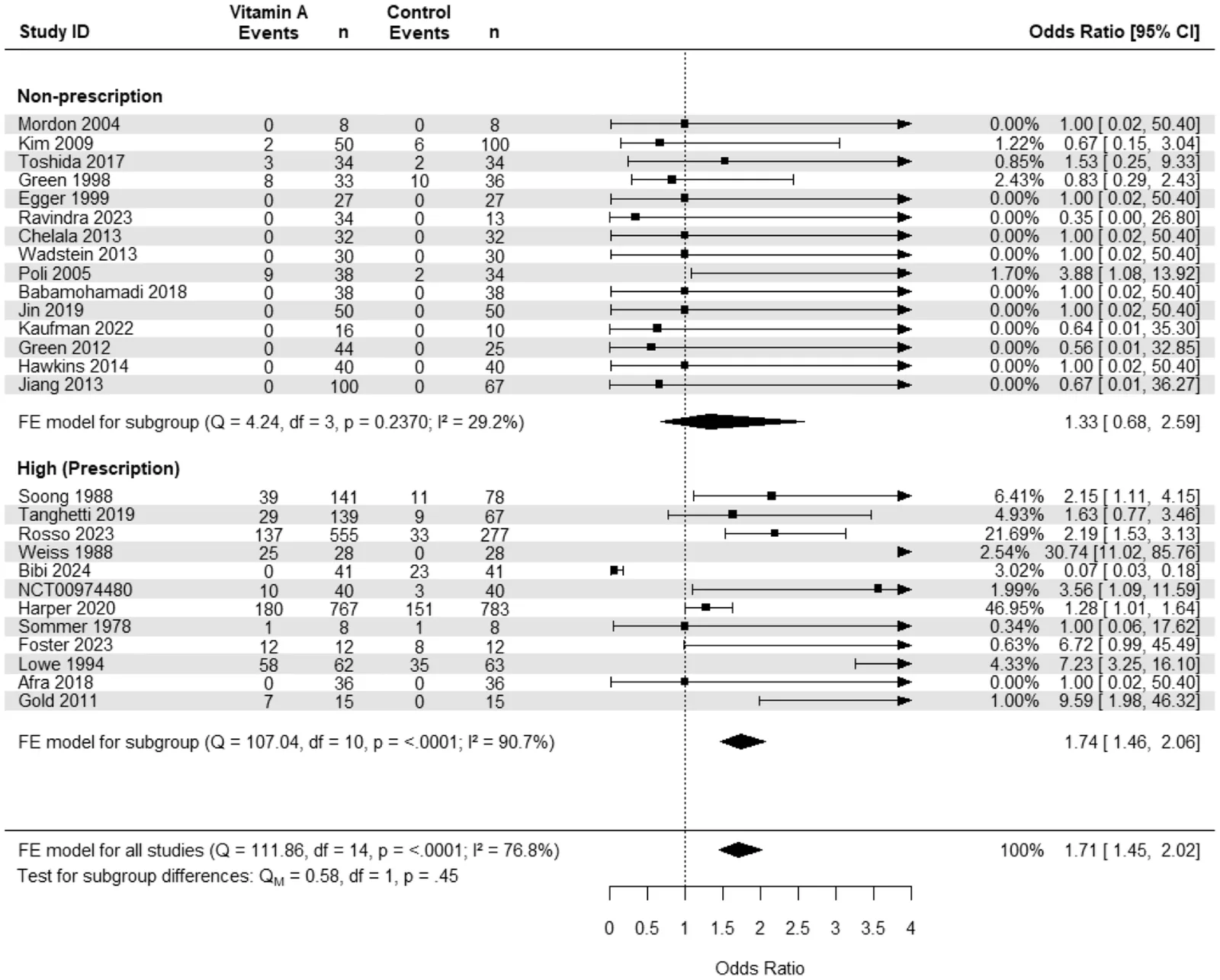
Non-prescription: low (*n* *=* 3 studies) or moderate (*n* *=* 1 study) non-prescription strength of OTC retinoids. Prescription: high-strength (*n* *=* 11 studies) prescription retinoids in the United States. On the *x*-axis, OR > 1 indicates greater odds of harm in the vit A group compared to control. ORs are plotted on the natural log scale. Studies with zero events across both arms are traditionally excluded from Peto odds; these results were included for transparency, even though they did not contribute to the meta-analysis.

Subgroup analysis found that high-strength (i.e., prescription) vit A derivatives were associated with 1.74 times the odds of any harm (95% CI, 1.46–2.06; *I*^2^ = 91%; 11 studies, 3220 participants); these included derivatives such as retinoic acid, tazarotene, tretinoin, and palovarotene. Conversely, low-strength (i.e., non-prescription) derivatives included retinol, retinol/retinyl palmitate/propionate/acetate, retinaldehyde, and non-specific vit A and did not have evidence of an association with increased odds of any harms (OR = 1.33; 95% CI, 0.68–2.59; *I*^2^ = 29%; four studies, 359 participants) (Fig. 3). The test for subgroup differences was not significant (*P* = 0.45).

### Clinical Outcomes

Important clinical outcomes were rarely the primary focus of the included trials. Most dermatologic studies were concerned with acne, photodamage, or cosmetic endpoints, whereas ocular surface outcomes were typically secondary measures. Supplementary Table S3 presents the extracted clinical outcomes. DED symptoms were reported in 13 studies. Although the heterogeneity of the measures, metrics, and time points prevented meta-analyses, these studies suggested small or negligible changes in symptoms, with no clear evidence of a consistent benefit or harm.^16^^,^^35^^–^^46^ Corneal staining was reported in 13 studies,^16^^,^^36^^–^^41^^,^^44^^–^^49^ and conjunctival staining was reported in three studies,^16^^,^^45^^,^^46^ but findings were heterogeneous and not easily pooled due to substantial variation in study design, patient populations, vit A formulations, application sites, and outcome measures. MG parameters were assessed in only two studies,^35^^,^^39^ of which only one provided usable data. One study evaluating vit A palmitate gel combined with cyclosporine A in patients with MGD-associated dry eye reported improvements in MG secretion characteristics after 12 weeks of treatment.^35^ The second study assessed MG morphology using meibography in patients with preoperative dry eye undergoing phacoemulsification and randomized patients to treatment with protein-free calf blood extract ophthalmic gel, sodium hyaluronate eye drops, or vit A palmitate eye gel. The study reported no significant differences in MG scores among treatment groups.^39^ Overall, findings did not demonstrate a consistent effect of vit A derivatives on DED severity. The limited data available were often secondary in nature, underpowered, and inconsistently reported.

## Discussion

Topical vit A containing products are widely used in dermatology, primarily for acne, photoaging, and other cosmetic applications, due to their ability to modulate sebocyte activity, normalize keratinocyte differentiation, and reduce inflammation.^18^^,^^26^ Given the close anatomic and functional parallels between sebaceous glands and MGs, it is plausible that topical vit A product application could influence eyelid and ocular surface health.^23^^,^^50^ This review highlights that, overall, topical vit A preparations are consistently associated with an increased incidence of local dermatological and ocular harms, including pain, dryness, burning, and redness.

These findings have important clinical implications. Dermatologists should be aware of potential spillover effects of periorbital retinoid use on ocular comfort and MG function, particularly in patients with existing or predisposing risk factors for DED. Optometrists and ophthalmologists should consider effects of topical retinoid use when evaluating patients with DED or MGD. Patient history should include dermatologic and cosmetic vit A product use near the eyelids. Consumers should also be aware that OTC retinol products may cause ocular or periorbital irritation. Prescription-strength agents such as tretinoin and tazarotene are more potent because they deliver retinoic acid directly, whereas OTC retinyl esters require enzymatic conversion and are generally less potent but not without risk, particularly near the eyes.^5^

Methodologically, this review highlights important gaps in the evidence base. Reporting of harms was inconsistent, with many studies omitting harms or failing to distinguish ocular from cutaneous outcomes. Few studies directly assessed MG outcomes, despite biological plausibility for retinoid-induced gland dysfunction based on evidence from potent oral vit A derivatives associated with MGD.^51^^,^^52^ In contrast, the limited available evidence evaluating topical ocular vit A formulations does not demonstrate a consistent adverse effect on MG structure or function. One study of vit A palmitate gel combined with cyclosporine A reported improvements in MG secretion characteristics in patients with MGD-associated dry eye, and another study evaluating vit A palmitate gel after phacoemulsification found no significant differences in meibography compared with other topical therapies.^35^^,^^39^ However, these findings are limited by the small number of studies, heterogeneous populations, and variability in MG outcome assessments. Ocular surface efficacy outcomes were also rarely prioritized and were often secondary or unmeasured in dermatology-focused trials. Additionally, reporting of participants’ underlying systemic and comorbidities was inconsistent across studies, limiting assessment of whether these conditions may have influenced outcomes. Together, these limitations hinder assessment of whether dermatologic benefits of vit A outweigh potential ocular risks.

This review has several limitations worth mentioning. Notably, this review was not designed to quantify efficacy outcomes, and the eligibility criteria may have excluded studies addressing these endpoints, limiting the ability to draw conclusions regarding effects on ocular surface outcomes. Although regulatory documents were planned to be examined for additional harms data, the volume and heterogeneity of products made this approach infeasible. Several factors were expected to potentially modify the risk of harms, including duration of exposure and location (i.e., directly to the eye, periorbitally, or elsewhere on the face), and these variables were extracted when reported. However, subgroup analyses based on these factors could not be performed because of substantial heterogeneity in exposure duration, which would have resulted in numerous subgroups, and poor reporting of application location. These factors may contribute to heterogeneity among studies included in the meta-analyses. If studies report these more consistently, future systematic reviews may be able to include these as factors in other analytical methods such as meta-regression to show how these affect the risk of harms. Finally, because title and abstract screening was stopped before the full set of records had been screened, some relevant studies may have been missed. However, this risk was considered minimal and unlikely to impact the findings, as the PICO Portal AI showed a 100% negative predictive value, indicating that all remaining records were predicted to be irrelevant based on the machine-learning model applied during the double-independent screening process.

Future research should prioritize rigorous designs that assess both efficacy and harms relevant to the ocular surface and MGs, using standardized measures of DED signs and symptoms and MG function. Improved harms reporting—particularly distinguishing ocular from dermatologic events—is essential for evidence-based recommendations.

## Conclusions

Although vit A–containing products remain valuable in dermatology and may confer select benefits to the cornea and conjunctiva, their use—particularly in the periorbital region—carries risk of ocular adverse effects and MGD. Clinicians should weigh these risks, counsel patients, and remain vigilant for ocular side effects. High-quality studies are needed to clarify dose–response relationships, compare OTC and prescription formulations, and determine whether dermatologic benefits meaningfully outweigh ocular harms.

## Supplementary Material

Supplement 1

Supplement 2

Supplement 3

Supplement 4

Supplement 5

Supplement 6

Supplement 7

Supplement 8

Supplement 9

Supplement 10

Supplement 11

## References

[1] Zhu G . Vitamin A and its derivatives–retinoic acid and retinoid pharmacology. *Am J Biomed Sci Res*. 2019; 3: 162–177.

[2] Pan J, Wang Q, Tu P. A topical medication of all-trans retinoic acid reduces sebum excretion rate in patients with forehead acne. *Am J Ther*. 2017; 24(2): e207–e212. 26872139 10.1097/MJT.0000000000000390

[3] Spierings NMK . Evidence for the efficacy of over-the-counter vitamin A cosmetic products in the improvement of facial skin aging: a systematic review. *J Clin Aesthet Dermatol*. 2021; 14(9): 33–40. 34980969 PMC8675340

[4] Szymański Ł, Skopek R, Palusińska M, et al. Retinoic acid and its derivatives in skin. *Cells*. 2020; 9(12): 2660. 33322246 10.3390/cells9122660 PMC7764495

[5] Connor MJ, Smit MH. The formation of all-trans-retinoic acid from all-trans-retinol in hairless mouse skin. *Biochem Pharmacol*. 1987; 36(6): 919–924. 3566789 10.1016/0006-2952(87)90185-7

[6] Chambon P . A decade of molecular biology of retinoic acid receptors. *FASEB J*. 1996; 10(9): 940–954. 8801176

[7] Orlandi A, Bianchi L, Costanzo A, Campione E, Spagnoli LG, Chimenti S. Evidence of increased apoptosis and reduced proliferation in basal cell carcinomas treated with tazarotene. *J Invest Dermatol*. 2004; 122(4): 1037–1041. 15102095 10.1111/j.0022-202X.2004.22414.x

[8] Kang S, Cho S, Chung JH, Hammerberg C, Fisher GJ, Voorhees JJ. Inflammation and extracellular matrix degradation mediated by activated transcription factors nuclear factor-κB and activator protein-1 in inflammatory acne lesions in vivo. *Am J Pathol*. 2005; 166(6): 1691–1699. 15920154 10.1016/s0002-9440(10)62479-0 PMC1602424

[9] Alam J, Yu Z, de Paiva CS, Pflugfelder SC. Retinoid regulation of ocular surface innate inflammation. *Int J Mol Sci*. 2021; 22(3): 1092. 33499199 10.3390/ijms22031092 PMC7866051

[10] Ubels JL, Foley K, Rismondo V. Retinol secretion by the lacrimal gland. *Invest Ophthalmol Vis Sci*. 1986; 27(8): 1261–1268. 3733370

[11] Samarawickrama C, Chew S, Watson S. Retinoic acid and the ocular surface. *Surv Ophthalmol*. 2015; 60(3): 183–195. 25890622 10.1016/j.survophthal.2014.10.001

[12] Gomes JAP, Azar DT, Baudouin C, et al. TFOS DEWS II iatrogenic report. *Ocul Surf*. 2017; 15(3): 511–538. 28736341 10.1016/j.jtos.2017.05.004

[13] Bron AJ, de Paiva CS, Chauhan SK, et al. TFOS DEWS II pathophysiology report. *Ocul Surf*. 2017; 15(3): 438–510. 28736340 10.1016/j.jtos.2017.05.011

[14] Tei M, Spurr–Michaud SJ, Tisdale AS, Gipson IK. Vitamin A deficiency alters the expression of mucin genes by the rat ocular surface epithelium. *Invest Ophthalmol Vis Sci*. 2000; 41(1): 82–88. 10634605

[15] Kim SW, Seo KY, Rhim T, Kim EK. Effect of retinoic acid on epithelial differentiation and mucin expression in primary human corneal limbal epithelial cells. *Curr Eye Res*. 2012; 37(1): 33–42. 22029601 10.3109/02713683.2011.620728

[16] Selek H, Ünlü N, Orhan M, Irkeç M. Evaluation of retinoic acid ophthalmic emulsion in dry eye. *Eur J Ophthalmol*. 2000; 10(2): 121–127. 10887922 10.1177/112067210001000205

[17] Nigam S, Gupta S, Gupta P. A randomized comparative trial of safety and efficacy of topical vitamin A and carboxymethylcellulose 1% on symptoms of dry eye disease after cataract surgery. *Ophthalmol J*. 2021; 6(0): 89–100.

[18] Aslan Bayhan S, Bayhan HA, Çölgeçen E, Gürdal C. Effects of topical acne treatment on the ocular surface in patients with acne vulgaris. *Cont Lens Anterior Eye*. 2016; 39(6): 431–434. 27422575 10.1016/j.clae.2016.06.009

[19] Caffery B, Josephson J. Ocular side effects of isotretinoin therapy. *J Am Optom Assoc*. 1988; 59(3): 221–224. 3258326

[20] Gilbert C . The eye signs of vitamin A deficiency. *Community Eye Health*. 2013; 26(84): 66. 24782581 PMC3936686

[21] Knop E, Knop N, Millar T, Obata H, Sullivan DA. The international workshop on meibomian gland dysfunction: report of the subcommittee on anatomy, physiology, and pathophysiology of the meibomian gland. *Invest Ophthalmol Vis Sci*. 2011; 52(4): 1938–1978. 21450915 10.1167/iovs.10-6997c PMC3072159

[22] Jones L, Craig JP, Markoulli M, et al. TFOS DEWS III: management and therapy report. *Am J Ophthalmol*. 2025; 279: 289–386. 40467022 10.1016/j.ajo.2025.05.039

[23] Ding J, Kam WR, Dieckow J, Sullivan DA. The influence of 13-cis retinoic acid on human meibomian gland epithelial cells. *Invest Ophthalmol Vis Sci*. 2013; 54(6): 4341–4350. 23722388 10.1167/iovs.13-11863 PMC3694789

[24] Mathers WD, Shields WJ, Sachdev MS, Petroll WM, Jester JV. Meibomian gland morphology and tear osmolarity: changes with Accutane therapy. *Cornea*. 1991; 10(4): 286–290. 1832371 10.1097/00003226-199107000-00002

[25] Chhadva P, Goldhardt R, Galor A. Meibomian gland disease: the role of gland dysfunction in dry eye disease. *Ophthalmology*. 2017; 124(11): S20–S26. 29055358 10.1016/j.ophtha.2017.05.031 PMC5685175

[26] Khalil S, Bardawil T, Stephan C, et al. Retinoids: a journey from the molecular structures and mechanisms of action to clinical uses in dermatology and adverse effects. *J Dermatolog Treat*. 2017; 28(8): 684–696. 28318351 10.1080/09546634.2017.1309349

[27] Market Scope. *Dry Eye Products Report: A Global Market Analysis for 2015 to 2021*. St. Louis: Market Scope; 2016.

[28] Abbott K, Qureshi R, Pucker A, Kruoch Z, Orgill M, Tichenor A. The safety of topically applied vitamin A ocularly and periorbitally: a systematic review protocol. Available at: https://www.crd.york.ac.uk/PROSPERO/view/CRD42024523189. Accessed August 21, 2026.

[29] Higgins JPT, Savović J, Page MJ, Elbers RG, Sterne JAC. Assessing risk of bias in a randomized trial. In: Higgins JPT, Thomas J, Chandler J, et al., eds. *Cochrane Handbook for Systematic Reviews of Interventions*. 2nd ed. New York: Wiley; 2019: 205–228.

[30] Liu S-H, Shaughnessy D, Leslie L, et al. Social determinants of dry eye in the United States: a systematic review. *Am J Ophthalmol*. 2024; 261: 36–53. 38242339 10.1016/j.ajo.2024.01.015 PMC11031303

[31] Deeks JJ, Higgins JP, Altman DG, Cochrane Statistical Methods Group. Analysing data and undertaking meta-analyses. In: Higgins JPT, Thomas J, Chandler J, et al., eds. *Cochrane Handbook for Systematic Reviews of Interventions*. 2nd ed. New York: Wiley; 2019: 241–284.

[32] Viechtbauer W . Conducting meta-analyses in R with the metafor package. *J Stat Softw*. 2010; 36(3): 1–48.

[33] Balduzzi S, Rücker G, Schwarzer G. How to perform a meta-analysis with R: a practical tutorial. *BMJ Ment Health*. 2019; 22(4): 153–160. 10.1136/ebmental-2019-300117 PMC10231495 31563865

[34] Schünemann HJ, Higgins JP, Vist GE, et al. Completing ‘summary of findings’ tables and grading the certainty of the evidence. In: Higgins JPT, Thomas J, Chandler J, et al., eds. *Cochrane Handbook for Systematic Reviews of Interventions*. 2nd ed. New York: Wiley; 2019: 375–402.

[35] Hao Y, Li S, Bao J, et al. Efficacy of 0.05% cyclosporine A combined with vitamin A palmitate in the treatment of meibomian gland dysfunction-related dry eye. *Chinese J Ophthalmol*. 2024; 60(2): 127–136. 10.3760/cma.j.cn112142-20231109-00221 38296318

[36] He Y, Hong W-W, Zhao H-Y. Vitamin A palmitate eye gel and carboxymethylcellulose sodium eye drops on prevention of dry eye after cataract surgery. *Int Eye Sci*. 2018: 1264–1267.

[37] Jin H-Y . Clinical observation of vitamin A palmitate eye gel combined with Tobramycin eye drops in repairing of corneal epithelial defect. *Int Eye Sci*. 2019; 19: 307–309.

[38] Kim EC, Choi J-S, Joo C-K. A comparison of vitamin a and cyclosporine a 0.05% eye drops for treatment of dry eye syndrome. *Am J Ophthalmol*. 2009; 147(2): 206–213.e3. 18848318 10.1016/j.ajo.2008.08.015

[39] Qi L, Jun W, Jia L. Effect of different drugs for ocular surface healing in patients with preoperative dry eye after phacoemulsification. *Int Eye Sci*. 2017; 17: 1700–1704.

[40] Lu Y-Y, Ren J, Ge X-H. Effect of vitamin a palmitate ophthalmic gel adjunctive therapy on tear film stability and inflammatory cytokines in patients with dry eye. *Int Eye Sci*. 2018; 18: 1135–1138.

[41] Badparva M, Veshagh M, Khosravi F, Mardani A, Ebrahimi H. Effectiveness of lubratex and vitamin A on ocular surface disorders in ICU patients: a randomized clinical trial. *J Intensive Care Soc*. 2021; 22(2): 136–142. 34025753 10.1177/1751143720912697 PMC8120574

[42] Ohashi Y, Watanabe H, Kinoshita S, Hosotani H, Umemoto M, Manabe R. Vitamin A eyedrops for superior limbic keratoconjunctivitis. *Am J Ophthalmol*. 1988; 105(5): 523–527. 3285694 10.1016/0002-9394(88)90245-0

[43] Schilling H, Koch J, Waubke T, Frank B. Treatment of the dry eye with vitamin A acid—an impression cytology controlled study. *Fortschr Ophthalmol*. 1989; 86(5): 530–534. 2583647

[44] Sun R, Yang M, Lin C, Wu Y, Sun J, Zhou H. A clinical study of topical treatment for thyroid-associated ophthalmopathy with dry eye syndrome. *BMC Ophthalmol*. 2023; 23(1): 72. 36803227 10.1186/s12886-023-02805-8 PMC9940084

[45] Ravindra AP, Sinha R, Bari A, et al. Retinol palmitate in management of chronic Steven–Johnson syndrome with ocular surface keratinization. *Ocul Surf*. 2023; 30: 160–167. 37689180 10.1016/j.jtos.2023.09.002

[46] Toshida H, Funaki T, Ono K, et al. Efficacy and safety of retinol palmitate ophthalmic solution in the treatment of dry eye: a Japanese phase II clinical trial. *Drug Des Devel Ther*. 2017; 11: 1871–1879. 10.2147/DDDT.S137825 PMC5491700 28694687

[47] Babamohamadi H, Nobahar M, Razi J, Ghorbani R. Comparing vitamin A and moist chamber in preventing ocular surface disorders. *Clin Nurs Res*. 2018; 27(6): 714–729. 29228795 10.1177/1054773817695618

[48] Sommer A . Treatment of corneal xerophthalmia with topical retinoic acid. *Am J Ophthalmol*. 1983; 95(3): 349–352. 6829680 10.1016/s0002-9394(14)78304-7

[49] Ebrahimi H, Badparva M. Effect of eye ointmet [sic] lubratex in the prevetion [sic] ocular surface disorders (OSD). Available at: https://trialsearch.who.int/Trial2.aspx?TrialID=IRCT2017040733278N1. Accessed August 21, 2026.

[50] Tsukada M, Schröder M, Orfanos CE, et al. 13-cis retinoic acid exerts its specific activity on human sebocytes through selective intracellular isomerization to all-trans retinoic acid and binding to retinoid acid receptors. *J Invest Dermatol*. 2000; 115(2): 321–327. 10951254 10.1046/j.1523-1747.2000.00066.x

[51] Zakrzewska A, Wiącek MP, Słuczanowska-Głąbowska S, Safranow K, Machalińska A. The effect of oral isotretinoin therapy on meibomian gland characteristics in patients with acne vulgaris. *Ophthalmol Ther*. 2023; 12(4): 2187–2197. 37301783 10.1007/s40123-023-00737-6 PMC10287853

[52] Düzgün E, Özkur E. The effect of oral isotretinoin therapy on meibomian gland morphology and dry eye tests. *J Dermatolog Treat*. 2022; 33(2): 762–768. 32506981 10.1080/09546634.2020.1774041

