# Supplementary material for: Potential Harms of Ocular and Periorbital Topical Vitamin A Application: A Systematic Review and Meta-Analysis
Source: Invest Ophthalmol Vis Sci. 2026 Sep 8;67(11):11. doi: 10.1167/iovs.67.11.11 (PMC13574213; doi:10.1167/iovs.67.11.11)
Supplement: Supplementary file 1 [file iovs-67-11-11_s001.docx]

**Supplementary Material**

**Cumulative loss of inner retinal neurons in healthy and glaucoma subjects**

Kazuhiro Kurokawa^1,*^, James A. Crowell^1^, Yan Liu^1^, Marcel Bernucci^1^, William H. Swanson^1^, and Donald T. Miller^1^

1. School of Optometry, Indiana University, Bloomington, IN 47405

Correspondence:

Kazuhiro Kurokawa, Discoveries in Sight Research Laboratories, Devers Eye Institute, Legacy Research Institute, Legacy Health, 1225 NE 2nd Ave, Portland, OR 97232, USA; [kkurokawa@deverseye.org](mailto:kkurokawa@deverseye.org)

Donald T. Miller; School of Optometry, Indiana University, 800 E Atwater Ave., Bloomington, IN 47405, USA; [dtmiller@iu.edu](mailto:dtmiller@iu.edu)

Current affiliations:

*Discoveries in Sight Research Laboratories, Devers Eye Institute, Legacy Research Institute, Legacy Health, Portland, Oregon, USA.

**Supplementary Notes:**

**Background Information for Histological Studies Listed in Table 4**

Gao and Hollyfield reported a 16% reduction in GC soma density across two age groups (second and sixth decades) each with three subjects.^1^ Assuming the subjects were either 20 or 60 years old and soma loss was constant over the 40-year period, we estimated their GC soma loss rate to be -0.37 %/yr (*P* < 0.05).

Blanks et al. counted GCL somas within a central 3-mm circle about the fovea and estimated a relatively high rate of GCL soma loss (-0.57 %/yr),^2^ the highest histologic estimate listed in Table 4. This rate was statistically significant (*P* = 0.016). Notably, their cohort consisted exclusively of older subjects (60-98 years), substantially older than those included in our study and the Gao and Hollyfield study.

Curcio and Drucker did not report a GCL soma loss rate, but we were able to estimate it from their data as well as a p-value.^3^ They counted GCL somas within a fovea-centric 10 mm circle in 9 normal donor eyes (age: 27-82 years old) and converted the cell counts to estimates of soma density. By performing linear regression on their GCL soma density estimates, we were able to estimate a GCL soma loss rate of -0.19%/yr, which was not statistically significant from zero (*P* > 0.05).

Harman et al. counted GCL somas within a central 10-mm circle in 12 normal eyes (age: 16-76 years old).^4^ Their reported GCL soma loss rate in the macula (-0.37 %/yr) was also not statistically significant (*P* = 0.28).

Other histological studies have reported age-dependent loss of GC axons in human^5–10^ and in monkey.^11–13^ While these studies demonstrated a decrease in axon number with age, only two, Jonas et al.^10^ and Balazsi et al.,^6^ reported a loss rate significantly different from zero. The results of Balazsi et al., however, were later questioned and the age effect was determined not to be significant after accounting for differences in tissue preparation.^9^ Jonas et al. included 56 normal eyes spanning a wide age range (19 to 88 years old) and reported an axon loss rate of -0.29 %/yr. However, when their analysis was restricted to subjects younger than 60 years (N = 26)—a range more comparable to our cohort—the loss rate was no longer significant, again suggesting that axon loss may accelerate with advancing age.

**References:**

1.	Gao H, Hollyfield JG. Aging of the human retina. Differential loss of neurons and retinal pigment epithelial cells. *Invest Ophthalmol Vis Sci*. 1992;33(1):1-17.

2.	Blanks JC, Torigoe Y, Hinton DR, Blanks RHI. Retinal pathology in Alzheimer’s disease. I. Ganglion cell loss in foveal/parafoveal retina. *Neurobiology of Aging*. 1996;17(3):377-384. doi:10.1016/0197-4580(96)00010-3

3.	Curcio CA, Drucker DN. Retinal ganglion cells in Alzheimer’s disease and aging. *Ann Neurol*. 1993;33(3):248-257. doi:10.1002/ana.410330305

4.	Harman A, Abrahams B, Moore S, Hoskins R. Neuronal density in the human retinal ganglion cell layer from 16–77 years. *The Anatomical Record*. 2000;260(2):124-131. doi:10.1002/1097-0185(20001001)260:2<124::AID-AR20>3.0.CO;2-D

5.	Dolman CL, McCormick AQ, Drance SM. Aging of the Optic Nerve. *Archives of Ophthalmology*. 1980;98(11):2053-2058. doi:10.1001/archopht.1980.01020040905024

6.	Balazsi AG, Rootman J, Drance SM, Schulzer M, Douglas GR. The Effect of Age on the Nerve Fiber Population of the Human Optic Nerve. *American Journal of Ophthalmology*. 1984;97(6):760-766. doi:10.1016/0002-9394(84)90509-9

7.	Johnson BM, Miao M, Sadun AA. Age-related decline of human optic nerve axon populations. *AGE*. 1987;10(1):5-9. doi:10.1007/BF02431765

8.	Repka MX, Quigley HA. The Effect of Age on Normal Human Optic Nerve Fiver Number and Diameter. *Ophthalmology*. 1989;96(1):26-32. doi:10.1016/S0161-6420(89)32928-9

9.	Mikelberg FS, Drance SM, Schulzer M, Yidegiligne HM, Weis MM. The Normal Human Optic Nerve: Axon Count and Axon Diameter Distribution. *Ophthalmology*. 1989;96(9):1325-1328. doi:10.1016/S0161-6420(89)32718-7

10.	Jonas JB, Schmidt AM, Müller-Bergh JA, Schlötzer-Schrehardt UM, Naumann GO. Human optic nerve fiber count and optic disc size. *Invest Ophthalmol Vis Sci*. 1992;33(6):2012-2018.

11.	Morrison JC, Cork LC, Dunkelberger GR, Brown A, Quigley HA. Aging changes of the rhesus monkey optic nerve. *Invest Ophthalmol Vis Sci*. 1990;31(8):1623-1627.

12.	Sandell JH, Peters A. Effects of age on nerve fibers in the rhesus monkey optic nerve. *Journal of Comparative Neurology*. 2001;429(4):541-553. doi:10.1002/1096-9861(20010122)429:4<541::AID-CNE3>3.0.CO;2-5

13.	Fortune B, Reynaud J, Cull G, Burgoyne CF, Wang L. The Effect of Age on Optic Nerve Axon Counts, SDOCT Scan Quality, and Peripapillary Retinal Nerve Fiber Layer Thickness Measurements in Rhesus Monkeys. *Trans Vis Sci Tech*. 2014;3(3):2-2. doi:10.1167/tvst.3.3.2

**Supplementary Figures:**

**Supplementary Figure S1.** Baseline clinical screening results for subject G142. The top row shows macular NFL, GCL and IPL thickness maps. The macular ganglion cell complex thickness (mGCCT) was defined as the combined thickness of the RNFL, GCL and IPL within the 6-mm-diameter macular region (outermost circle). The mean thickness of each layer was estimated by dividing its measured volume (mm^3^), reported in the top row, by the area of the 6-mm-diameter circle area (π r^2^, where r = 3 mm). The mGCCT was then calculated as the sum of the estimated mean thicknesses of the NFL, GCL, and IPL. The bottom row shows the corresponding Humphrey 24-2 visual field test results.

**Supplementary Figure S2.** Baseline clinical screening results for subject G147. The top row shows macular NFL, GCL and IPL thickness maps. The mGCCT was estimated from the measured volumes of the NFL, GCL, and IPL, as reported in the top row, using the mGCCT definition described in the caption of Supplementary Fig. 1. The bottom row shows the corresponding Humphrey 24-2 visual field test results.

**Supplementary Figure S3.** Procedural flowchart for determining GCL soma loss. G1-4 are graders who manually identified and counted the GCL somas independently for each year (top row). G5-7 are graders who determines the number of somas lost between baseline and each follow-up year (middle rows). Finally, G8 combines the intermediary soma results to determine the soma loss rate (bottom row). ***N*** is the total GCL somas present within sub-region. ***L_Y0-#_*** is the number of GCL soma lost between T0 and T1 (Y0-1), T0 and T2 (Y0-2), and T0 and T3 (Y0-3), respectively.

**Supplementary Figure S4**. Longitudinal AO-OCT imaging in subject H053. (a) Spectralis SLO image showing the retinal locations imaged with AO-OCT (red boxes). Labels indicate retinal eccentricities relative to the fovea, with the optic nerve head (ONH) shown on right. (b) GCL soma mosaics at seven retinal eccentricities acquired at baseline (T0), 1 year (T1), 2 year (T1) and 3 years (T3) after baseline. Yellow boxes indicate the regions used for analysis. Blue arrows point to unusually bright somas. White scale bars denote 50 µm.

**Supplementary Figure S5**. Longitudinal AO-OCT imaging in subject H116. (a) Spectralis SLO image showing the retinal locations imaged with AO-OCT (red boxes). Labels indicate retinal eccentricities relative to the fovea, with the optic nerve head (ONH) shown on right. (b) GCL soma mosaics at five retinal eccentricities acquired at baseline (T0), 1 year (T1), 2 year (T1) and 3 years (T3) after baseline. Yellow boxes indicate the regions used for analysis. Red arrows point to microcysts within the GCL that change in size over the course of the study. White scale bars denote 50 µm.

**Supplementary Figure S6**. Longitudinal AO-OCT imaging in subject H125. (a) Spectralis SLO image showing the retinal locations imaged with AO-OCT (red boxes). Labels indicate retinal eccentricities relative to the fovea, with the optic nerve head (ONH) shown on right. (b) GCL soma mosaics at five retinal eccentricities acquired at baseline (T0), 1 year (T1), 2 year (T1) and 3 years (T3) after baseline. Yellow boxes indicate the regions used for analysis. White scale bars denote 50 µm.

**Supplementary Figure S7**. Longitudinal AO-OCT imaging in subject H127. (a) Spectralis SLO image showing the retinal locations imaged with AO-OCT (red boxes). Labels indicate retinal eccentricities relative to the fovea, with the optic nerve head (ONH) shown on right. (b) GCL soma mosaics at five retinal eccentricities acquired at baseline (T0), 1 year (T1), 2 year (T1) and 3 years (T3) after baseline. Yellow boxes indicate the regions used for analysis. White scale bars denote 50 µm.

**Supplementary Figure S8**. Longitudinal AO-OCT imaging in subject H154. (a) Spectralis SLO image showing the retinal locations imaged with AO-OCT (red boxes). Labels indicate retinal eccentricities relative to the fovea, with the optic nerve head (ONH) shown on right. (b) GCL soma mosaics at five retinal eccentricities acquired at baseline (T0) and 1 year after baseline (T1). Yellow boxes indicate the regions used for analysis. White scale bars denote 50 µm.

**Supplementary Figure S9**. Longitudinal AO-OCT imaging in subject H155. (a) Spectralis SLO image showing the retinal locations imaged with AO-OCT (red boxes). Labels indicate retinal eccentricities relative to the fovea, with the optic nerve head (ONH) shown on right. (b) GCL soma mosaics at five retinal eccentricities acquired at baseline (T0) and 1 year after baseline (T1). Yellow boxes indicate the regions used for analysis. White scale bars denote 50 µm.

**Supplementary Figure S10.** Longitudinal assessment of glaucomatous damage in subject G147. (a) GCL thickness maps measured with Spectralis OCT at baseline (left) and two years later (middle). The corresponding retinal thickness change over this interval is shown at right. (b) AO-OCT composite C-scan and B-scan images (left) indicating the imaged retinal regions, with magnified views of the examined locations at baseline (middle) and two years later (right). Green and blue boxes denote the retinal regions imaged by AO-OCT at baseline and at the 2-year follow-up, respectively. The yellow dashed box denotes the retinal location analyzed for soma loss. White scale bars denote 100 µm.

**Supplementary Table S1** Grader information

| Retinal location | Subject ID | Grader 1  (T0) | Grader 2  (T1) | Grader 3  (T2) | Grader 4  (T3) | Grader 5  (T0-T1) | Grader 6  (T0-T2) | Grader 7  (T0-T3) |
| --- | --- | --- | --- | --- | --- | --- | --- | --- |
|  |  |  |  |  |  |  |  |  |
| 12–13.5˚T | H127 | A7 | NaN^*^ | A2 | AS | A2 | A2 | A5 |
|  | H116 | A1 | A2 | A8 | AS | A2 | A6 | A5 |
|  | H125 | A1 | A3 | A5 | AS | A2 | A2 | A2 |
|  | H053 | A1 | A2 | A5 | AS | A2 | A2 | A5 |
|  | H154 | AS | AS | - | - | A2 | - | - |
|  | H155 | AS | AS | - | - | A2 | - | - |
|  |  |  |  |  |  |  |  |  |
| 8–9.5˚T | H127 | A1 | A4 | A5 | AS | A2 | A2 | A6 |
|  | H116 | A1 | A3 | A6 | AS | A2 | A2 | A5 |
|  | H125 | A1 | A4 | A5 | AS | A2 | A2 | A2 |
|  | H053 | A1 | A3 | A6 | AS | A2 | A2 | A8 |
|  | H154 | AS | AS | - | - | A2 | - | - |
|  | H155 | AS | AS | - | - | A2 | - | - |
|  |  |  |  |  |  |  |  |  |
| 6–7.5˚T | H127 | A1 | A4 | A5 | AS | A2 | A2 | A6 |
|  | H116 | A1 | A4 | A8 | AS | A2 | A2 | A6 |
|  | H125 | A1 | A3 | A6 | AS | A2 | A2 | A2 |
|  | H053 | A1 | A4 | A5 | AS | A2 | A2 | A5 |
|  | H154 | AS | AS | - | - | A2 | - | - |
|  | H155 | AS | AS | - | - | A2 | - | - |
|  |  |  |  |  |  |  |  |  |
| 3–4.5˚T | H127 | A1 | A4 | A8 | AS | A2 | A6 | A5 |
|  | H116 | A1 | A4 | A7 | AS | A2 | A2 | A5 |
|  | H125 | A1 | A3 | AS | AS | A2 | A2 | A2 |
|  | H053 | A1 | A4 | A5 | AS | A2 | A6 | A5 |
|  | H154 | AS | AS | - | - | A2 | - | - |
|  | H155 | AS | AS | - | - | A2 | - | - |
|  |  |  |  |  |  |  |  |  |
| 1.25–2.75˚T | H127 | A1 | A4 | A6 | AS | A2 | A2 | A5 |
|  | H116 | A1 | A4 | A7 | AS | A2 | A6 | A5 |
|  | H125 | A1 | A3 | A6 | AS | A2 | A2 | A2 |
|  | H053 | A1 | A3 | A7 | AS | A2 | A2 | A6 |
|  | H154 | AS | AS | - | - | A2 | - | - |
|  | H155 | AS | AS | - | - | A2 | - | - |
|  |  |  |  |  |  |  |  |  |
| 1.25–2.75˚N | H053 | A1 | A3 | A5 | AS | A2 | A2 | A5 |
| 3–4.5˚N | H053 | A1 | A2 | A5 | AS | A2 | A2 | A6 |

A1-8 are individual human agents (trained graders) assigned to manually identify and count somas.

AS is a software agent assigned to identify and count somas.

^*^12°-13.5° retinal location of Subject H127 was not analyzed at T1 due to insufficient image overlap with T0.

**Supplementary Table S2** Summary of the AO-OCT measurements

| Retinal location | Subject ID | Imaged location (degrees) | | | GCL soma density (counts/mm^2^) | GCL thickness (µm) | GCL soma loss rate (%/yr) |
| --- | --- | --- | --- | --- | --- | --- | --- |
|  |  | Radial | Horizontal | Vertical |  |  |  |
| 12–13.5˚ T | H127 | 11.50 | 11.15 | 2.82 | 4572 | 13.5 | -0.031 |
|  | H116 | 12.63 | 12.60 | 0.84 | 5934 | 18.9 | -0.076 |
|  | H125 | 12.66 | 12.63 | 0.87 | 6091 | 16.1 | -0.063 |
|  | H053 | 12.02 | 11.67 | 2.88 | 4109 | 15.6 | -0.073 |
|  | H154^*^ | 11.82 | 11.82 | 0.29 | 5482 | 18.9 | -0.077 |
|  | H155^*^ | 12.03 | 12.02 | 0.44 | 4492 | 15.0 | 0^†^ |
|  | Mean +/- SD | 12.11±0.46 | 11.98±0.57 | 1.36±1.18 | 5114±831 | 16.3±2.2 | -0.06±0.02 |
|  |  |  |  |  |  |  |  |
| 8–9.5˚ T | H127 | 8.28 | 8.25 | 0.70 | 12898 | 28.4 | -0.097 |
|  | H116 | 9.20 | 9.12 | 1.19 | 14916 | 31.2 | -0.149 |
|  | H125 | 9.11 | 9.06 | 0.90 | 13635 | 30.7 | -0.054 |
|  | H053 | 7.84 | 7.84 | 0.12 | 11914 | 27.9 | -0.074 |
|  | H154^*^ | 8.68 | 8.68 | 0.09 | 11335 | 29.0 | 0^†^ |
|  | H155^*^ | 8.48 | 8.48 | 0.15 | 9588 | 26.9 | -0.232 |
|  | Mean +/- SD | 8.6±0.51 | 8.57±0.49 | 0.52±0.47 | 12381±1864 | 29±1.7 | -0.12±0.07 |
|  |  |  |  |  |  |  |  |
| 6–7.5˚ T | H127 | 6.60 | 6.56 | 0.67 | 16185 | 32.1 | -0.216 |
|  | H116 | 7.27 | 7.20 | 1.02 | 21253 | 41.6 | -0.163 |
|  | H125 | 7.10 | 7.09 | 0.41 | 18452 | 36.9 | -0.154 |
|  | H053 | 6.23 | 6.21 | 0.49 | 16066 | 37.8 | 0^†^ |
|  | H154^*^ | 7.12 | 7.12 | 0.03 | 17151 | 37.9 | 0^†^ |
|  | H155^*^ | 6.36 | 6.36 | 0.20 | 16836 | 37.1 | 0^†^ |
|  | Mean +/- SD | 6.78±0.44 | 6.76±0.43 | 0.47±0.35 | 17657±1959 | 37.2±3 | 0.18±0.03 |
|  |  |  |  |  |  |  |  |
| 3–4.5˚ T | H127 | 3.53 | 3.51 | 0.38 | 27206 | 48.7 | -0.095 |
|  | H116 | 4.41 | 4.21 | 1.31 | 34750 | 58.1 | -0.090 |
|  | H125 | 4.03 | 4.01 | 0.44 | 31103 | 52.9 | -0.130 |
|  | H053 | 3.08 | 3.08 | 0.15 | 27780 | 56.2 | -0.128 |
|  | H154^*^ | 3.90 | 3.89 | 0.20 | 29063 | 61.1 | -0.185 |
|  | H155^*^ | 2.90 | 2.88 | 0.38 | 25093 | 52.0 | -0.223 |
|  | Mean +/- SD | 3.64±0.58 | 3.6±0.53 | 0.47±0.42 | 29166±3385 | 54.8±4.5 | -0.14±0.05 |
|  |  |  |  |  |  |  |  |
| 1.5–3˚ T | H127 | 2.42 | 1.77 | 1.66 | 31303 | 54.8 | -0.040 |
|  | H116 | 2.25 | 1.74 | 1.42 | 26751 | 46.8 | -0.189 |
|  | H125 | 2.12 | 2.09 | 0.32 | 26851 | 44.0 | -0.061 |
|  | H053 | 1.82 | 1.80 | 0.26 | 27994 | 53.2 | -0.157 |
|  | H154^*^ | 2.15 | 2.15 | 0.06 | 23855 | 52.8 | -0.092 |
|  | H155^*^ | 2.01 | 2.00 | 0.15 | 19536 | 41.6 | 0^†^ |
|  | Mean +/- SD | 2.13±0.21 | 1.93±0.18 | 0.64±0.7 | 26048±3994 | 48.9±5.5 | -0.11±0.06 |
|  |  |  |  |  |  |  |  |
| 1.5–3˚ N | H053 | 2.19 | -2.18 | -0.23 | 28693 | 52.0 | -0.165 |
| 3–4.5˚ N | H053 | 3.72 | -3.72 | -0.20 | 33443 | 62.9 | -0.085 |
| 4-7˚ NI | G142 | 6.37 | -4.41 | -4.59 | 1326 | 12.7 | -0.26 |
| 4-7˚ NI | G147 | 5.12 | -3.46 | -3.78 | 1527 | 21.9 | -31.6 |

^*^Loss rate was calculated using a single follow-up measurement relative to baseline.

^†^No measured loss of GCL somas.

**Supplementary Videos:**

**Video S1.** Animation demonstrating the use of the customized graphic user interface to identify and mark GCL somas. The interface displays simultaneous en face (XY) and cross sectional (XZ and YZ B-scan) views of an example AO-OCT volume. The grader analyzes the entire GCL volume, identifying each soma and its 3D coordinates based on its three-dimensional appearance. This process is facilitated by moving the 3D yellow crosshairs (cursor position) in real time to the volumetric center of each soma. Each identified soma is marked with a red ‘x.’ The soma’s 3D size is then measured, and a 3D oval of the same size is added to the AO-OCT volume. Each oval is assigned a random color to distinguish adjacent somas. Color ovals are then superimposed onto the interface displays, which helps confirm that (1) their center coordinates and size estimation are reasonable and (2) there are no missing or overlapping somas.

**Video S2.** Animation demonstrating the image alternation technique to facilitate detection of soma loss. The technique was incorporated into the customized graphic user interface. Each soma was independently assessed using the alternation method with the depth position optimized for that soma. Somas identified at baseline and at the 1-year follow-up are marked with red and green ‘x’ symbols, respectively. For a visualization purpose, these symbols are superimposed onto the interface display after projecting them along the depth. Displaying a difference between two coordinates facilitates the process for detecting soma loss while correcting errors.

**Video S3.** Time-lapse view of GCL somas imaged in subject H053 at seven retinal locations across four timepoints. The top row shows AO-OCT B-scan images, and the bottom row shows corresponding en face images at baseline and at the 1-, 2-, and 3-year follow-up visits.

**Video S4.** Time-lapse view of GCL somas imaged in subject H116 at five retinal locations across four timepoints. The top row shows AO-OCT B-scan images, and the bottom row shows corresponding en face images at baseline and at the 1-, 2-, and 3-year follow-up visits.

**Video S5.** Time-lapse view of GCL somas imaged in subject H125 at five retinal locations across four timepoints. The top row shows AO-OCT B-scan images, and the bottom row shows corresponding en face images at baseline and at the 1-, 2-, and 3-year follow-up visits.

**Video S6.** Time-lapse view of GCL somas imaged in subject H127 at five retinal locations across four timepoints. The top row shows AO-OCT B-scan images, and the bottom row shows corresponding en face images at baseline and at the 1-, 2-, and 3-year follow-up visits.

**Video S7.** Time-lapse view of GCL somas imaged in subject H154 at five retinal locations across two timepoints. The top row shows AO-OCT B-scan images, and the bottom row shows corresponding en face images at baseline and at the 1-year follow-up visit.

**Video S8.** Time-lapse view of GCL somas imaged in subject H155 at five retinal locations across two timepoints. The top row shows AO-OCT B-scan images, and the bottom row shows corresponding en face images at baseline and at the 1-year follow-up visit.

**Video S9.** Example of a GCL soma loss event at 1.5–3° retinal eccentricity in subject H053. The loss occurs at the yellow cursor position between baseline and the 1-year follow-up visit.

**Video S10.** Example of a GCL soma migration event at 3–4.5° retinal eccentricity in subject H053. The upward (anterior) migration occurs at the yellow cursor position between the 2- and 3-year follow-up visits.
